# Periodized carbohydrate intake influences metabolic flexibility and indices of running economy during endurance training in recreationally active males

**DOI:** 10.3389/fnut.2025.1750042

**Published:** 2026-01-15

**Authors:** Anna Maria Kripp, Andreas Feichter, Daniel König

**Affiliations:** 1 Department of Nutritional Sciences, Faculty of Life Sciences, University of Vienna, Vienna, Austria; 2 Vienna Doctoral School of Pharmaceutical, Nutritional and Sport Sciences, University of Vienna, Vienna, Austria; 3 Department of Sport Science, Centre for Sport Science and University Sports, University of Vienna, Vienna, Austria

**Keywords:** endurance performance, high carbohydrate diet, long-term carbohydrate periodization, low-carb-high-fat diet, recreational runners, substrate use

## Abstract

**Purpose:**

Limited research has explored the long-term impact of diet periodization on metabolism, performance, and body composition. This eight-week study examined the effects of long-term periodized carbohydrate (CHO) intake on performance, substrate utilization, and body composition in recreationally active males.

**Methods:**

Twenty-four runners (V̇O_2_ peak: 51 ± 8 mL·min^−1^·kg^−1^) were randomly assigned to one nutritional intervention: (1) PER: 4 weeks of ≤ 50 g CHO per day followed by 4 weeks of high CHO; (2) LCHF: 8 weeks of ≤ 50 g CHO per day; or (3) CHO: 8 weeks of high CHO. All subjects underwent the same endurance training. Performance and substrate use were measured using a graded exercise test at baseline (T-0), after 4 weeks (T-1), and after 8 weeks (T-2). Body weight and composition were measured using bioelectrical impedance analysis.

**Results:**

While performance improved over time, no significant differences were found between groups. However, running economy at lactate threshold only improved in the PER group (*p* < 0.05). Fat oxidation increased from T-0 to T-1 in PER and LCHF group (*p* < 0.001), accompanied by a decrease in CHO oxidation at the lactate threshold (*p* < 0.001). In the LCHF group, no further changes occurred, whereas in the PER group fat oxidation decreased and CHO oxidation increased from T-1 to T-2 (*p* < 0.001). Both PER and LCHF group lost weight and fat mass from T-0 to T-1, with further losses in the LCHF group (*p* < 0.001).

**Conclusion:**

A LCHF diet increased fat oxidation while reducing carbohydrate utilization during exercise. Reintroduction of carbohydrates restored substrate utilization patterns, indicating reversible changes in metabolic flexibility. However, no clear effects on performance outcomes were observed. These findings suggest that periodized carbohydrate intake primarily influences metabolic responses rather than performance, and further research using performance-based assessments is warranted. These adaptations appear to be predominantly metabolic and physiological in nature.

**Clinical trial registration:**

https://doi.org/10.17605/OSF.IO/VM7KB.

## 1 Introduction

The principle of periodization is already deeply embedded in sport science for decades (1, 2). However, in addition to the purposeful sequencing of individual training units to structure training load and intensity, it is important to consider the impact of nutrition on training adaptations and performance. Early guidelines already emphasized aligning energy and macronutrient intake with different training phases (3). Since then, the term periodized nutrition has been defined as the “planned, purposeful, and strategic use of specific nutritional interventions to enhance adaptations targeted by individual exercise sessions or periodic training plans” (4). When implementing such strategies, it is crucial to consider individual energy requirements to prevent symptoms associated with low energy availability. Moreover, it is essential to differentiate between periods of training and competition when implementing these adjustments (5).

The objective of prolonged endurance exercise is to delay fatigue in order to maintain performance at a high level for as long as possible (6). From a metabolic perspective, ATP resynthesis during endurance exercise depends on exercise intensity, training status and nutritional state (7, 8). Therefore, oxidative phosphorylation of fat and carbohydrates (CHO) represents the primary energy supply during endurance exercise (9). As CHO stores in muscle and liver are limited, maximizing fat oxidation may help preserve endogenous CHO availability. In recent years, there has been growing interest in the potential benefits of a low-carbohydrate, high-fat (LCHF) diet to enhance fat oxidation and reduce CHO utilization (10). It has been shown that adherence to a LCHF diet increases fat oxidation and might induce weight loss in some athletes (11). However, prolonged CHO restriction may also lead to performance losses and decreased metabolic flexibility or adverse health-related outcomes, including dysregulated blood lipids, increased inflammatory responses or impaired iron metabolism (12–14).

There is evidence suggesting that periodized CHO intake may influence training adaptions by regulating muscle cellular signaling pathways (15, 16). Accordingly, several fueling strategies have been proposed that temporarily reduce CHO availability, including sleep-low, fasted exercise or twice-a-day training (17). Some studies have reported increased performance following the implementation of these approaches (18, 19). However, a recent systematic review and meta-analyses concluded that CHO periodization does not consistently improve performance compared with a high CHO diet (20). Given the limited number of studied and heterogenous intervention protocols, further research is warranted.

Therefore, the present study aimed to investigate whether periodizing CHO intake over an eight-week training period provides advantages compared with a continous high-carbohydrate or low-carbohydrate diet. Outcomes included performance in a graded exercise test, substrate metabolism and body composition in recreationally active males. We hypothesized that a LCHF diet during the initial base phase would increase fat oxidation and reduce CHO utilization, whereas subsequent CHO reintroduction during intensified training would restore metabolic flexibility.

## 2 Methods

### 2.1 Study design

**Figure 1:**
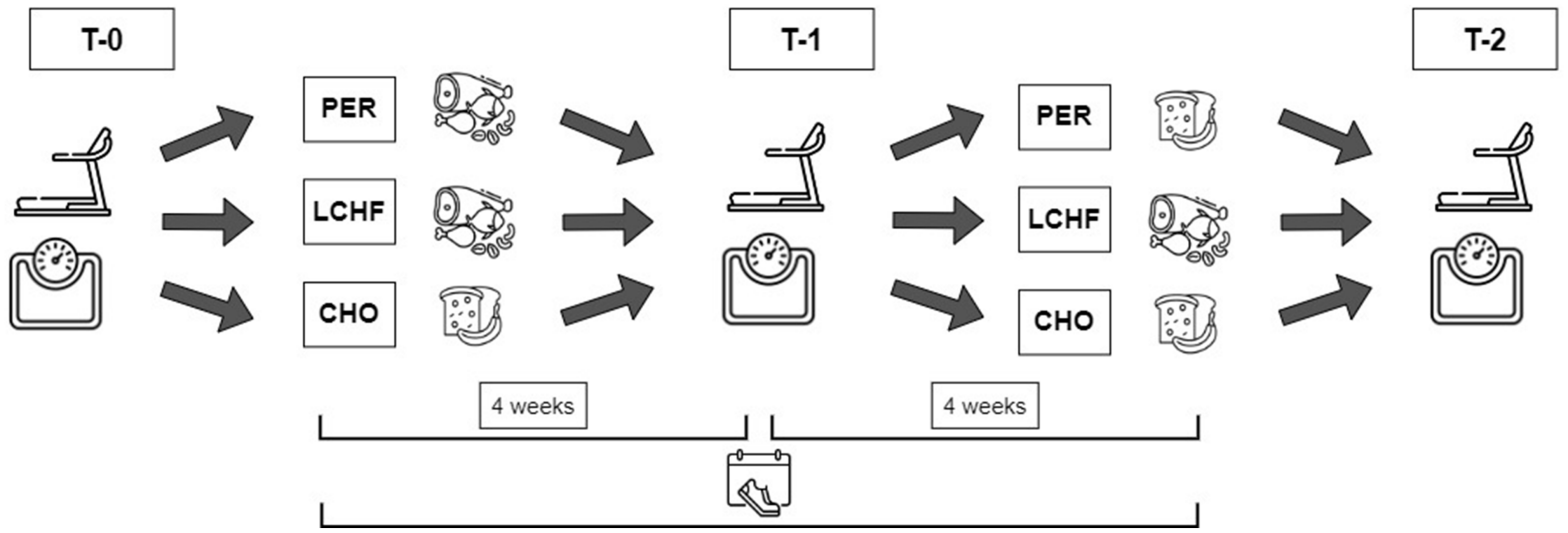
Overview of the study schedule. T-0: Before the study, T-1: after 4 weeks, T-2: after 8 weeks, PER, periodized CHO intake; LCHF, low-CHO-high fat diet; CHO, high CHO diet.

The presented study was designed as a controlled, randomized, non-blinded study and combined a training and nutritional intervention over a time period of 8 weeks. Participants were recruited primarily through advertisements on various social media platforms and at the sports university center. The study was approved by the Ethical Committee of the University of Vienna (Reference Number: 01081), registered at Open Science Framework^1^ and conducted in accordance with the Declaration of Helsinki. Prior to participation all subjects gave their written informed consent. A comprehensive overview of the study is depicted in Figure 1.

### 2.2 Participants

**Figure 2:**
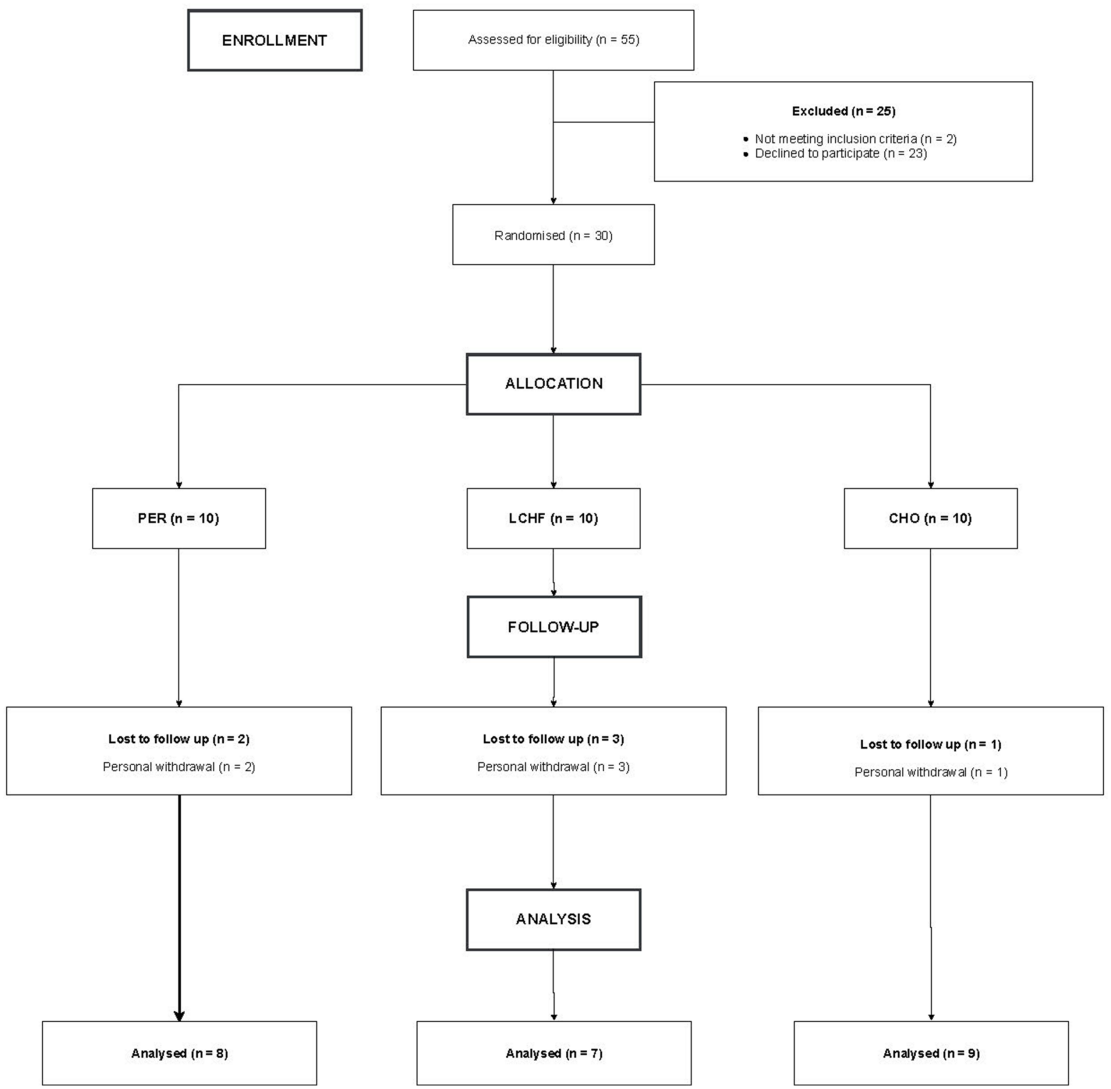
Study flow chart.

As demonstrated in Figure 2, the flow chart outlining the subject recruitment, randomization and follow-up process reveals that, of the 55 subjects who registered for the trial, 30 were randomly allocated to one of the nutritional intervention groups. Non-randomized subjects were excluded on the basis that they did not meet the inclusion criteria or due to personal withdrawal. The inclusion criteria encompassed individuals between the ages of 18 and 40 years, meeting the criteria for recreationally active individuals (participating in two to three training sessions per week), and those deemed to be ready for physical activity (as per the Physical Activity Readiness Questionnaire). Exclusion criteria included previous experience with a LCHF diet in the last 6 months, contraindications to physical activity according to the American College of Sports Medicine Guidelines (21), the use of medications or dietary supplements, chronic diseases, and arterial hypertension.

An *a priori* power analysis for the repeated measurements design was conducted using G*Power (Version 3.1.9.7 for Windows) for a two-way repeated-measures ANOVA with a within–between interaction (group × time). The calculation was based on changes in time to exhaustion, using effect sizes derived from previous studies with comparable interventions and outcome (22, 23). An effect size of 0.160 with a power of 0.8, alpha error probability of 0.05 and the correlation among measurements of 0.9 resulted in a total sample size of 21. With a surcharge for dropouts, 10 participants per group would be sufficient to attain significant results and ensure adequate power.

### 2.3 Measurements

Following an initial medical screening, participants underwent anthropometric and demographic data collection. Body composition was measured using bioelectric impedance (Seca mBCA 514/515, seca GmbH & Co. KG, Hamburg, Germany) and training status was assessed. Prior to randomization (T-0), after 4 weeks (T-1) and after 8 weeks (T-2), a graded exercise test was performed on a treadmill (Quasar med, h/p/cosmos sports & medical GmbH, Nussdorf-Traunstein, Germany) starting at 6 km·h^−1^ with increasing speed by 1.5 km·h^−1^ every 3 min until exhaustion. For all tests, participants arrived in the laboratory in a fed state and at the same time of the day (± 1 h) to avoid circadian influences. To ensure a comparable nutritional status, nutrition was standardized for the last 24 h before the test and adjusted to the nutritional intervention.

During the exercise testing capillary blood samples were collected at baseline, at the end of each step, and at the point of exhaustion. These samples were analyzed using the Biosen lactate analyzer (Biosen S-line, KF-diagsnotic, Barleben/Magdeburg, Germany). The respective lactate concentrations were analyzed using Ergonizer software (Freiburg, Germany) and used to individually adjust training zones for the training intervention. The first lactate threshold (LT) was defined as the earliest moment of an increase in blood lactate concentration with increasing exercise intensity (24, 25). As glycogen availability changed due to the nutritional intervention, the modified D_max_ method was used to compare changes at the second lactate threshold (26). D_max mod_ was calculated by the Ergonizer software, which identifies the point of maximal perpendicular distance between the lactate–intensity curve and a straight line connecting the initial and final lactate values (27, 28). Both LT and D_max mod_ are reported as corresponding running speeds. In addition, running speed at fixed blood lactate concentrations of 2 mmol·L^−1^ (RS 2 mmol·L^−1^) and 4 mmol·L^−1^ (RS 4 mmol·L^−1^) was determined from the lactate–speed relationship.

Furthermore, oxygen uptake (V̇O_2_) and carbon dioxide output (V̇CO_2_) were measured continuously by means of a breath-by-breath gas analyzer (MetaLyzer 3B, Cortex Biophysik GmbH, Leipzig, Germany), and then the respiratory exchange ratio (RER) was calculated as the ratio between V̇O2 and V̇CO2. Prior to each experimental session, the device was calibrated in accordance with the manufacturer’s instructions. V̇O_2_ peak was defined as the 30 s rolling average maximum V̇O_2_ value during the test. The subjects were then randomly assigned to the intervention groups in order to minimize any baseline differences, as suggested by Hopkins (29). Heart rate was measured throughout the test using a heart rate belt (Polar H10, Polar Electro OY, Kempele, Finland).

### 2.4 Calculations

From the graded exercise test, peak running speed (PRS), time to exhaustion (TTE), substrate metabolism and running economy (RE) were calculated. If the final increment of the graded exercise test could not be completed, the PRS was calculated as proposed by Kuipers et al. (30):


$$\mathrm{PRS}=V\;\mathrm{com}+\frac{t}{3}*1.5$$

in which v _com_ is the last increment completed, and *t* the number of minutes the final not completed speed was sustained. TTE was calculated as the total duration of the test from the first increment (6 km·h^−1^) on.

To calculate substrate metabolism, the average values of V̇O_2_ (L·min^−1^) and RER from the last minute of each 3-min exercise stage were used. Up to RER = 1.00, fat and carbohydrate oxidation were calculated using stochiometric equations according to Péronnet and Massicotte (31):


$$\dot{V}CO_{2}[L\cdot \mathrm{mi}n^{-1}]=\mathrm{RER}\times \dot{V}O_{2}[L\cdot \mathrm{mi}n^{-1}]$$

$$\begin{array}{l} \mathrm{Fat}\;\text{oxidation}[g\cdot \mathrm{mi}n^{-1}]=1.695\times \dot{V}O_{2}[L\cdot \mathrm{mi}n^{-1}]\\ -1.701\times \dot{V}CO_{2}[L\cdot \mathrm{mi}n^{-1}] \end{array}$$

$$\begin{array}{l} \text{Carbohydrate oxidation}[g\cdot \mathrm{mi}n^{-1}]=4.585\times \dot{V}CO_{2}[L\cdot \mathrm{mi}n^{-1}]\\ -3.2255\times \dot{V}O_{2}[L\cdot \mathrm{mi}n^{-1}] \end{array}$$

Maximal fat oxidation (MFO) was defined as the highest rate of fat oxidation observed during the incremental exercise test, while Fat max represented the exercise intensity at which MFO occurred in % of V̇O_2_ peak.

The calculation of total energy expenditure (TEE) was performed by adding fat and carbohydrate oxidation of all stages, where RER was below 1.00. Further, to assess and compare the parameters over the duration of the graded exercise test, for fat and carbohydrate oxidation and blood lactate concentration the area under the curve (AUC) was calculated between the start of the test and the final increment with RER below 1.00 or the final increment completed (t_n_) using the following equation:


$$\mathrm{AU}C_{0-t_{n}}=\frac{1}{2}\sum_{i=0}^{t_{n}}(t_{i+1}-t_{i})x(C_{i}+C_{i+1})$$

where t_n_ is the final completed increment, t_i_ the km·h^−1^ of the increment at which the parameter was measured and C_i_ the respective concentration/value to that timepoint.

Lastly, relative V̇O_2_ in mL·kg^−1^ and running speed corresponding to the lactate threshold (LT) at each respective time point were used to calculate RE, thereby ensuring that measurements were obtained at the lactate threshold and within the moderate-intensity domain to minimize the influence of the V̇O₂ slow component. Running speed at LT was chosen to ensure that running economy was assessed at a comparable relative exercise intensity at the lactate threshold for all participants, thereby avoiding inter-individual differences in metabolic domain classification. This approach is consistent with established definitions of running economy and previous methodological recommendations (32, 33). Additionally, RE at a fixed speed of 7.5 km·h^−1^ was calculated.

### 2.5 Nutritional intervention

Following comprehensive nutritional training and under the supervision of trained dieticians, participants were randomly assigned to one of the three dietary interventions:

- PER: 4-week regimen with ≤ 50 g CHO per day and more than 60% of daily energy intake from fats directly followed by 4-week regimen comprising 50–60% of daily energy intake from CHO.
- LCHF: 8-week regimen with ≤ 50 g CHO per day with more than 60% of daily energy intake from fats.
- CHO: 8-week regimen comprising 50–60% of daily energy intake from CHO.

The subjects followed the dietary guidelines under free-living conditions, preparing their own meals to accommodate personal preferences. Food choices were made independently while adhering to the assigned dietary framework. Regular contact with dietitians was maintained to ensure adherence and provide immediate support for any questions. A detailed overview of the nutritional guidelines can be found in the Supplementary material.

Adherence to the nutritional intervention was assessed via the FDDB app (https://fddb.mobi/ in the free version), a widely used nutrition tracking app that provides an extensive food database and allows barcode scanning of packaged foods to improve accuracy of nutrient information. Participants were instructed to record all food and beverage intake throughout 3 days per week (two weekdays and one weekend day) in real time. To enhance data quality and compliance, participants were asked to weekly provide screenshots of their dietary records, which were reviewed by the study team for completeness and plausibility. After completion of the study, participants exported their full dietary logs directly from the application and submitted them for analysis.

Dietary records were screened for missing entries, implausible energy intakes, and inconsistencies. When unclear entries were identified, participants were contacted for clarification whenever possible.

Further, a food frequency questionnaire (FFQ) was completed at three time points: during the four-week monitoring period prior to the intervention (T-0), after the first 4 weeks of the intervention (T-1), and after completion of the eight-week intervention (T-2). Therefore, the validated DEGS1-FFQ was used. It collects the frequency and quantity of 53 food items eaten in the last 4 weeks (34). The FFQ was completed online and converted into the nutritional intake according to previous proposed methods (35). The mean of the data of the dietary logs and the FFQ was calculated and used for further calculations.

Furthermore, in the PER and LCHF group, urine test strips (Ketostix, Acensia Diabetes Care Austria GmbH, Vienna, Austria) were utilized to assess ketosis. Consequently, prior to the commencement of the training intervention, participants of the PER and LCHF groups were instructed to initiate the low CHO diet and to test ketone bodies on a daily basis. The training intervention was only initiated when subjects were in ketosis (> 0.5 mmol·L^−1^). During the intervention, ketone bodies were measured once weekly for the duration of the low CHO intervention. The participants were provided with detailed instructions for the test stripes. It was essential that the test was conducted using morning urine.

### 2.6 Exercise intervention

The endurance training intervention was designed to align with the periodized nutritional intervention. However, the same program was completed by all subjects. Thus, the initial 4 weeks of the training plan emphasized basic endurance, while the subsequent 4 weeks focused on interval sessions. During the four-week periods, the intensity and duration of the sessions increased progressively from week 1 to 3, with week 4 designated as a compensation week. The graded exercise test was conducted during this week. Each week of the training program consisted of five running sessions in total. Compliance with the training program was assessed via the subjects’ sports watches, as the training sessions were completed individually. Prior to the commencement of the intervention period, the subjects were instructed on the correct sequence of sessions and the required duration of rest between each session.

The duration of the session was consistent for all test subjects, while the intensity was based on the individually determined training zones from the graded exercise test. Basic endurance sessions were heart rate based and interval sessions tempo based. The training intervention and explanations to the training zones are provided in the Supplementary material.

Throughout the intervention phase, general condition, gastrointestinal well-being and perceived exertion were assessed daily using a visual analogue scale (VAS) (36).

### 2.7 Statistical analyses

Statistical analyses were performed using Statistical Package for the Social Sciences Software (SPSS for Windows, Version 28, SPSS Inc., Chicago, IL) and figures were created using GraphPad Prism (GraphPad Prism Version 8.0.2 for Windows, GraphPad Software, San Diego, California, USA). The level of significance was set a *α* = 0.05. The results of the descriptive analysis are shown as mean ± standard deviation (SD).

Normality assumptions were assessed based on the residuals of the ANOVA models using the Shapiro–Wilk test. Baseline differences between groups were analyzed using one-way ANOVA. When assumptions for parametric testing were not met, the Kruskal–Wallis test was applied.

To asses time (within subject factor), group (between subject factor) and time x group interaction effects, a two-way mixed ANOVA with Bonferroni-corrected *post hoc* analysis was used. In case of a significant interaction effect, simple main effects for time and group were analyzed. Effect sizes (ηp^2^) for all interaction effects are displayed.

A post hoc power analysis was conducted using G*Power (Version 3.1.9.7 for Windows) for the group x time interaction of the two-way mixed ANOVA. The analysis was based on the observed partial eta squared values of peak running speed, time to exhaustion and relative V̇O_2_ peak, and an alpha level of 0.05, a total sample size of 24 participants, three groups, and the number of repeated measurements.

## 3 Results

### 3.1 Study population

In total 24 of the initial 30 randomized subjects completed the study and were included into the statistical analysis (8 subjects in PER, 7 subjects in LCHF and 9 subjects in CHO). The reasons for an early withdrawal from the study are shown in the flow chart (Figure 2). They were not attributable to any study measurement or the intervention.

The baseline characteristics are displayed in Table 1. Baseline characteristics are presented descriptively as mean ± SD. No statistical comparisons were performed at baseline in accordance with CONSORT recommendations for randomized controlled trials (37, 38). In accordance with the set inclusion criteria, they engaged in physical activity on 3 ± 1 days per week. With a mean V̇O_2_ peak of 51 ± 8 mL·min^−1^·kg^−1^, they conformed to the target demographic.

**Table 1:** Baseline characteristics.

	PER	LCHF	CHO
*N*	8	7	9
Age [years]	30 ± 6	29 ± 3	30 ± 4
Height [cm]	184 ± 6	180 ± 8	181 ± 6
Weight [kg]	84 ± 10	80 ± 12	74 ± 7
BMI [kg·min^−2^]	24.9 ± 3.2	24.8 ± 2.2	22.6 ± 1.6
Active days per week	3 ± 1	3 ± 1	3 ± 1
V̇O_2_ peak [mL·min^−1^·kg^−1^]	48 ± 9	50 ± 5	53 ± 9

The investigation revealed that the analysis of the daily filled VAS scale demonstrated no significant differences between the groups or across the weeks. Consequently, the general condition, gastrointestinal well-being, and perceived exhaustion levels were found to be comparable between the groups.

Ketone bodies measurement revealed good compliance with the nutritional intervention. After implementing the low CHO diet in the PER and LCHF group mean ketone body concentration in the first week was 0.8 ± 1.0 mmol·L^−1^. During the training intervention mean ketone body concentration for both groups in the first 4 weeks was 1.9 ± 1.4 mmol·L^−1^ and in the second 4 weeks 1.3 ± 1.5 mmol·L^−1^ in the LCHF group.

### 3.2 Nutritional intervention

Participant compliance with dietary recording was high. All 24 participants submitted complete dietary records for the entire study period, including exported food logs from the dietary tracking application. Regular screenshot submissions further indicated consistent use of the dietary tracking app throughout the intervention.

Energy and macronutrient intake before and during the intervention are summarized in Table 2. Where a significant group × time interaction was detected, follow-up simple main effects analyses were performed and are reported below. At baseline (T-0), no significant differences were observed between groups for total energy intake or macronutrient intake (all *p* > 0.05). Total energy intake did not differ significantly between groups or across timepoints.

**Table 2:** Energy and macronutrient intake before and during the intervention.

	Group	Pre Intervention	First intervention period	Second intervention period	Time × Group (ηp^2^)
Energy intake [kcal]	PER	2033 ± 354	2044 ± 428	1979 ± 492	0.615 (0.060)
	LCHF	1791 ± 385	1771 ± 321	1,679 ± 326	
	CHO	1993 ± 350	2,252 ± 368	2,142 ± 370	
Carbohydrate intake [%]	PER	44.3 ± 7.7 ^2^	10.9 ± 3.2 ^1,3, c^	44.8 ± 7.9 ^2, b^	**< 0.001 (0.882)**
	LCHF	43.3 ± 2.7 ^2,3^	8.9 ± 2.6 ^1, c^	10.3 ± 2.8 ^1, a,c^	
	CHO	46.5 ± 7	48.3 ± 6.3 ^a,b^	48.7 ± 6.4 ^b^	
Carbohydrate intake [g·day^−1^]	PER	224 ± 47 ^2^	55 ± 18 ^1,3, c^	222 ± 69 ^2, b^	**< 0.001 (0.763)**
	LCHF	194 ± 41 ^2,3^	39 ± 10 ^1, c^	44 ± 15 ^1, a,c^	
	CHO	232 ± 53	275 ± 70 ^a,b^	264 ± 64 ^b^	
Fat intake [%]	PER	33.8 ± 6.0 ^2^	59.9 ± 5.6 ^1,3, c^	34.4 ± 5.3 ^2, b^	**< 0.001 (0.815)**
	LCHF	35.5 ± 2.8 ^2,3^	63.2 ± 3.2 ^1, c^	62.2 ± 4.3 ^1, a,c^	
	CHO	32.3 ± 5.5	32.6 ± 7.7 ^a,b^	30.9 ± 6.4 ^b^	
Fat intake [g·day^−1^]	PER	77 ± 22 ^2^	136 ± 34 ^1,3, c^	76 ± 23 ^2, b^	**< 0.001 (0.470)**
	LCHF	71 ± 18 ^2,3^	125 ± 26 ^1, c^	116 ± 26 ^1, a,c^	
	CHO	72 ± 19	81 ± 20 ^a,b^	72 ± 14 ^b^	
Protein intake [%]	PER	18.3 ± 6.9 ^2^	26.8 ± 4.6 ^1,3, c^	18.3 ± 3.6 ^2, b^	**< 0.001 (0.688)**
	LCHF	17.6 ± 2.4 ^2,3^	26.8 ± 2.9 ^1, c^	26.6 ± 3.5 ^1, a,c^	
	CHO	19.0 ± 2.6	17.6 ± 2.2 ^a,b^	18.0 ± 2.5 ^b^	
Protein intake [g·day^−1^]	PER	91 ± 28	136 ± 34 ^3, c^	90 ± 25 ^2^	**0.002 (0.331)**
	LCHF	78 ± 15 ^2,3^	118 ± 21 ^1^	110 ± 15 ^1^	
	CHO	94 ± 20	99 ± 18 ^a^	95 ± 13	

Bold numbers represent significant *p*-values. T-0: before the study, T-1: mean intake week 1–4, T-2: mean intake week 5–8. ^1,2,3^ significantly different intake between the timepoints during the intervention: ^1^compared to pre intervention, ^2^compared to first intervention period, ^3^compared to second intervention period. ^a,b,c^ significantly different intake between groups during the intervention: ^a^compared to PER, ^b^compared to LCHF, ^c^compared to CHO.

#### 3.2.1 Carbohydrate intake

In the PER group, relative and absolute CHO intake decreased significantly from T-0 to T-1 (−33.4 ± 6.5%, *p* < 0.001; −168 ± 39 g·day^−1^, *p* < 0.001) and increased again from T-1 to T-2 (+34.0 ± 9.1%, *p* < 0.001; +167 ± 74 g·day^−1^, *p* = 0.001). In the LCHF group, CHO intake decreased significantly from T-0 to T-1 (−34.5 ± 3.0%, *p* < 0.001; −155 ± 36 g·day^−1^, *p* < 0.001) and remained similarly reduced at T-2 compared with T-0 (−33.1 ± 2.6%, *p* < 0.001; −150 ± 36 g·day^−1^, *p* < 0.001), with no further change between T-1 and T-2 (*p* > 0.05). No significant changes in CHO intake were observed in the CHO group (*p* > 0.05).

Between-group comparisons showed that at T-1, relative and absolute CHO intake were significantly higher in the CHO group compared with the PER and LCHF group (*p* < 0.001; ηp^2^ = 0.946 and 0.617, respectively). At T-2, CHO intake was significantly higher in the PER and CHO group compared with the LCHF group (*p* < 0.001; ηp^2^ = 0.892 and 0.754, respectively).

#### 3.2.2 Fat intake

A similar pattern was observed for fat intake. In the PER group, relative and absolute fat intake increased significantly from T-0 to T-1 (+26.0 ± 7.9%, *p* < 0.001; +59 ± 41 g·day^−1^, *p* < 0.001) and decreased again from T-1 to T-2 (−25.5 ± 9.5%, *p* < 0.001; −61 ± 33 g·day^−1^, *p* = 0.004). In the LCHF group, fat intake increased significantly from T-0 to T-1 (+27.6 ± 3.3%, *p* < 0.001; +54 ± 21 g·day^−1^, *p* = 0.002) and remained elevated until T-2 (*p* > 0.05). No significant changes were observed in the CHO group (*p* > 0.05).

During the first 4 weeks (T-1), relative and absolute fat intake were significantly higher in the PER and LCHF group compared with the CHO group (*p* < 0.001; ηp^2^ = 0.863 and 0.335, respectively). During the second 4 weeks (T-2), fat intake was significantly higher in the LCHF group compared with both the PER and CHO group (*p* < 0.001; ηp^2^ = 0.875 and 0.485, respectively).

#### 3.2.3 Protein intake

In the PER group, relative protein intake increased significantly from T-0 to T-1 (+8.5 ± 5.3%, *p* < 0.001) and decreased again from T-1 to T-2 (−8.5 ± 3.1%, *p* < 0.001). Absolute protein intake in this group decreased significantly during the second half of the intervention (−46 ± 4 g·day^−1^, *p* = 0.010). In the LCHF group, relative and absolute protein intake increased from T-0 to T-1 (+9.1 ± 2.1%, *p* < 0.001; +40 ± 24 g·day^−1^, *p* = 0.014) and remained stable until T-2 (*p* > 0.05). No significant changes in protein intake were observed in the CHO group (*p* > 0.05).

At T-1, relative protein intake was significantly higher in the PER and LCHF group compared with the CHO group (*p* < 0.001; ηp^2^ = 0.665).

### 3.3 Exercise intervention

No differences in training minutes were found neither in total (PER: 1674 ± 148 min, LCHF: 1553 ± 201 min, CHO: 1517 ± 159 min *p* = 0.121), nor when splitting the minutes in interval (PER: 523 ± 45 min, LCHF: 507 ± 60 min, CHO: 463 ± 91 min, *p* = 0.222) or steady-state (PER: 1151 ± 154 min, LCHF: 1046 ± 147 min, CHO: 1054 ± 174 min, *p* = 0.371) sessions.

### 3.4 Endurance performance

Neither relative or absolute V̇O_2_ peak, nor peak running speed or time to exhaustion showed a significant time x group interaction (Table 3). Post-hoc power analysis for the interaction effects yielded achieved power values of 0.59 for PRS, 0.75 for TTE and 0.89 for V̇O_2_ peak based on the observed effect sizes.

**Table 3:** Changes in endurance performance.

	Group	T-0	T-1	T-2	Time × Group (ηp^2^)
Relative V̇O_2_ peak [mL·min^−1^·kg^−1^]	PER	48 ± 9	49 ± 7	49 ± 8	0.313 (0.105)
	LCHF	50 ± 5	52 ± 6	53 ± 5	
	CHO	53 ± 9	52 ± 9	54 ± 9	
Absolute V̇O_2_ peak [mL·min^−1^]	PER	3,986 ± 474	3,930 ± 392	3,979 ± 420	0.900 (0.025)
	LCHF	4,012 ± 579	3,952 ± 571	3,959 ± 624	
	CHO	3,908 ± 498	3,773 ± 480	3,905 ± 508	
PRS [km·h^−1^]	PER	15.5 ± 1.8	16.1 ± 1.4	16.4 ± 1.0	0.643 (0.057)
	LCHF	15.4 ± 1.6	15.7 ± 1.4	16.3 ± 1.4	
	CHO	16.3 ± 2.5	16.6 ± 2.6	16.9 ± 2.4	
TTE [sec]	PER	1,502 ± 210	1,591 ± 169	1,613 ± 186	0.485 (0.077)
	LCHF	1,485 ± 189	1,526 ± 171	1,593 ± 163	
	CHO	1,599 ± 298	1,633 ± 310	1,673 ± 290	
RE at LT [mL·kg^−1^·km^−1^]	PER	200 ± 31 ^3^	197 ± 15	173 ± 29 ^1^	**0.007 (0.282)**
	LCHF	199 ± 23	201 ± 24	204 ± 27	
	CHO	207 ± 30	190 ± 33	207 ± 29	
RE at 7.5 km·h^−1^ [mL·kg^−1^·km^−1^]	PER	220 ± 23	210 ± 13	198 ± 23	0.053 (0.195)
	LCHF	229 ± 12	226 ± 13	234 ± 15	
	CHO	221 ± 19	213 ± 24	221 ± 27	

Bold numbers represent significant *p*-values. ^1,2,3^ significantly different intake between the timepoints during the intervention: ^1^compared to T-0, ^2^compared to T-1, ^3^compared to T-2.

Despite the absence of significant interaction effects, significant main effects of time were observed for PRS and TTE (*p* < 0.001, respectively), indicating changes over the course of the study independent of group allocation. Specifically, PRS increased significantly from T-0 to T-1 (+0.4 ± 0.4 km·h^−1^, *p* < 0.001) and improved further from T-1 to T-2 (+0.4 ± 0.5 km·h^−1^, *p* = 0.003). Similarly, TTE increased significantly from T-0 to T-1 (+55 ± 76 s, *p* = 0.005), with an additional significant improvement from T-1 to T-2 (+42 ± 77 s, *p* = 0.045).

Running economy at lactate threshold showed a significant time x group interaction (*p* = 0.007). Simple main effects of time revealed that in the PER group RE decreased significantly from T-0 to T-2 (−27 ± 21 in mL·kg^−1^ km^−1^, *p* = 0.026). No further significant changes were observed. To address potential confounding effects of changes in lactate threshold velocity, running economy was additionally analyzed at a fixed running speed of 7.5 km·h^−1^ across all time points. While no significant group × time interaction was observed (*p* = 0.053), descriptive data indicated a progressive reduction in oxygen cost in the PER group, whereas no consistent changes were observed in the LCHF or CHO groups (Table 3).

### 3.5 Body composition

**Figure 3:**
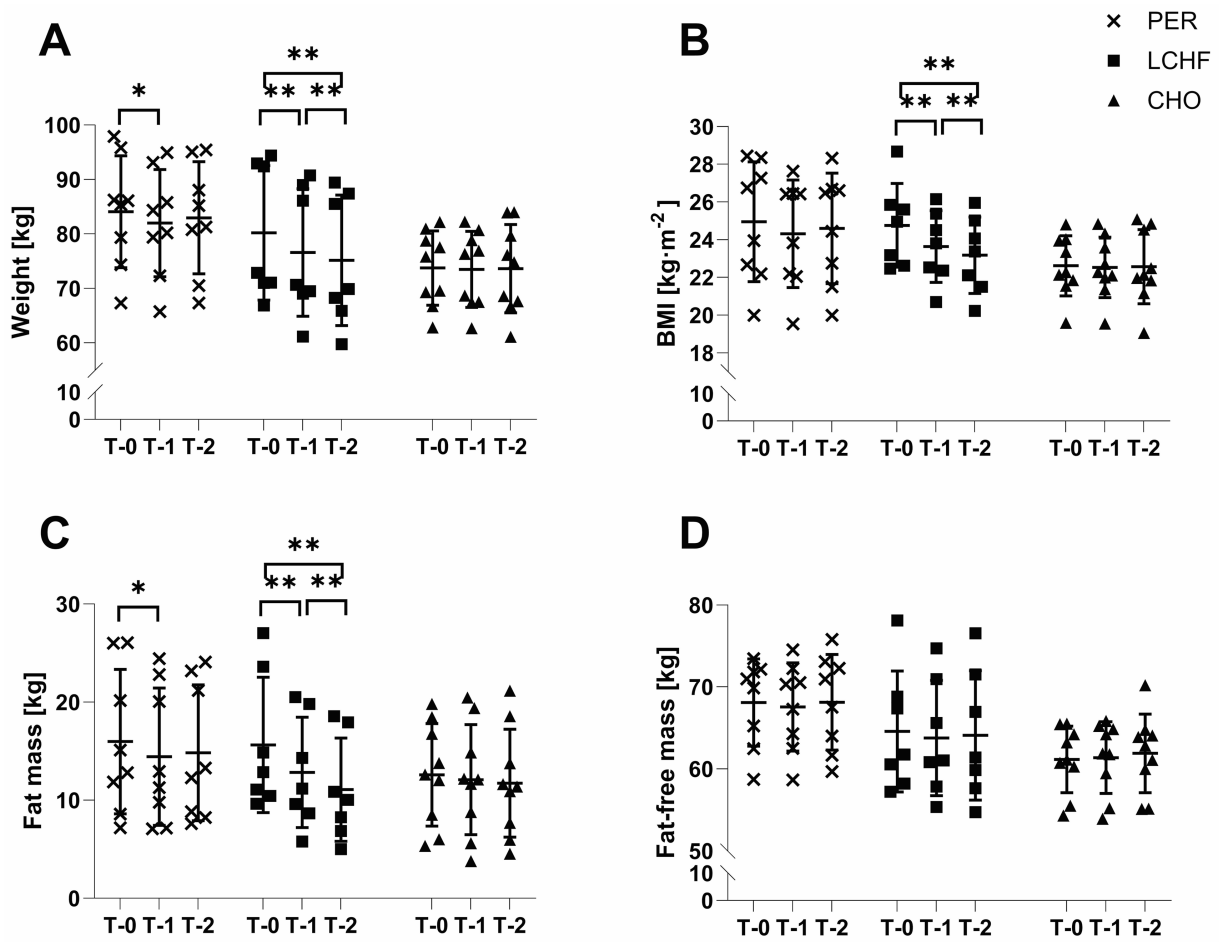
Changes in body composition over time. Individual data points and group means ± SD are shown for the PER (×), LCHF (■), and CHO (▲) groups at baseline (T-0), week 4 (T-1), and week 8 (T-2). **(A)** Weight, **(B)** BMI, **(C)** Fat mass, and **(D)** Fat-free mass. Asterisks indicate significant time effects (*p* < 0.05, *p < 0.001).

Body weight (*p* < 0.001, ηp^2^ = 0.426), BMI (*p* < 0.001, ηp^2^ = 0.424), and fat mass (*p* < 0.001, ηp^2^ = 0.414) showed significant interaction effects. However, no differences between the groups were observed at any timepoint. In the PER group weight and fat mass decreased significantly from T-0 to T-1 (−2.1 ± 1.9 kg, *p* = 0.048, −1.5 ± 1.0 kg, *p* = 0.013) without any further change until T-2. In the LCHF group weight, BMI and fat mass showed a significant decrease from T-0 to T-1 (*p* < 0.001) with an even further decrease until T-2 (*p* < 0.001, Figure 3). For fat-free mass no significant interaction (*p* > 0.05, ηp^2^ = 0.071) effect was found.

### 3.6 Metabolic outcomes

**Figure 4:**
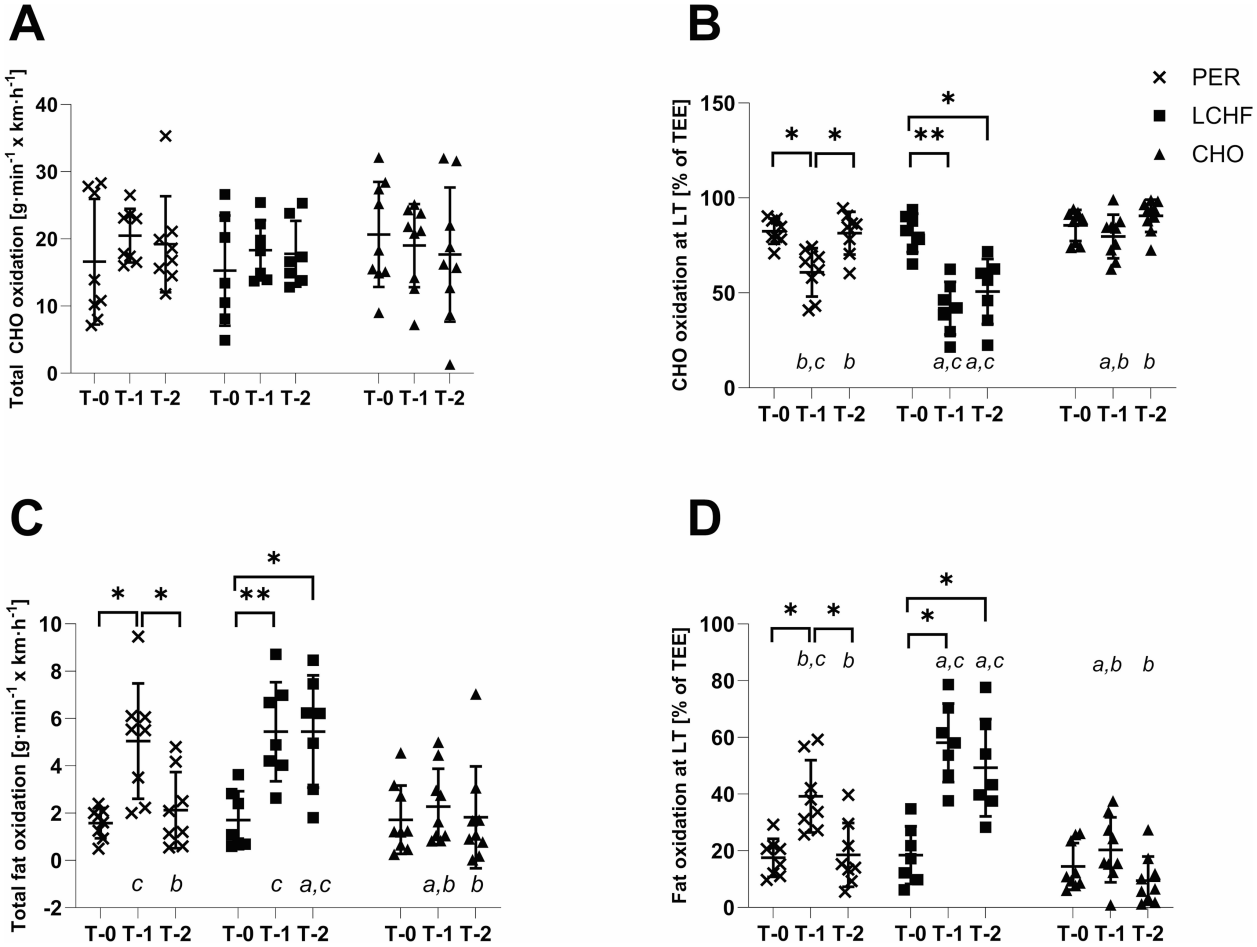
Changes in substrate metabolism over time. Individual data points and group means ± SD are shown for the PER (×), LCHF (■), and CHO (▲) groups at baseline (T-0), week 4 (T-1), and week 8 (T-2). **(A)** Total carbohydrate (CHO) oxidation, **(B)** CHO oxidation at the lactate threshold (LT), **(C)** total fat oxidation, and **(D)** fat oxidation at LT. Asterisks indicate significant time effects (*p* < 0.05, *p < 0.001). Lowercase letters indicate significant between-group differences at the respective time points: a compared with PER, b compared with LCHF, c compared with CHO.

Regarding substrate metabolism, both fat oxidation (*p* < 0.001, ηp^2^ = 0.427) and maximum fat oxidation (MFO) showed significant group x time interaction effects (Figure 4; Table 4). Follow-up simple main effects analyses are reported below.

**Table 4:** Change in substrate metabolism.

	Group	T-0	T-1	T-2	Time × Group (ηp^2^)
MFO [g·min^−1^]	PER	0.4 ± 0.1 ^2^	0.8 ± 0.2 ^1,3, c^	0.4 ± 0.2 ^2, b^	**<0.001 (0.473)**
	LCHF	0.4 ± 0.2 ^2,3^	0.8 ± 0.1 ^1, c^	0.8 ± 0.2 ^1, a,c^	
	CHO	0.4 ± 0.1	0.4 ± 0.2 ^a,b^	0.3 ± 0.22 ^b^	
Fat max [% of V̇O_2_ peak]	PER	50 ± 11	60 ± 14	55 ± 10	0.315 (0.104)
	LCHF	54 ± 13	68 ± 13	66 ± 6	
	CHO	51 ± 12	48 ± 17	56 ± 16	
TEE [g·min^−1^]	PER	13.4 ± 6.0	17.9 ± 4.9	14.9 ± 5.5	**0.034 (0.215)**
	LCHF	12.0 ± 6.2 ^3^	16.7 ± 4.9	16.3 ± 5.0 ^1^	
	CHO	15.7 ± 5.9	14.7 ± 4.8	13.7 ± 7.6	
AUC lactate [mmol·L^−1^ x km·h^−1^]	PER	53.9 ± 11.5	47.3 ± 12.2	51.8 ± 8.0	0.530 (0.071)
	LCHF	55.4 ± 10.2	44.6 ± 10.5	48.8 ± 7.7	
	CHO	49.4 ± 11.4	47.7 ± 14.2	49.2 ± 13.4	
RS at LT [km·h^−1^]	PER	8.2 ± 1.8	8.8 ± 2.0	8.9 ± 2.0	0.289 (0.110)
	LCHF	7.9 ± 1.9	8.4 ± 1.9	9.0 ± 2.1	
	CHO	8.9 ± 2.1	9.2 ± 2.3	9.4 ± 2.1	
RS at D_max mod_ [km·h^−1^]	PER	12.4 ± 1.6	12.4 ± 2.7	13.3 ± 1.5	0.852 (0.031)
	LCHF	12.4 ± 1.6	12.7 ± 1.6	13.3 ± 1.4	
	CHO	13.0 ± 2.3	13.5 ± 2.4	13.8 ± 2.2	
RS at 2 mmol·L^−1^ [km·h^−1^]	PER	9.7 ± 2.4 ^2,3^	11.3 ± 2.6 ^1^	11.1 ± 2.2 ^1^	**0.007 (0.278)**
	LCHF	9.2 ± 1.9 ^2,3^	11.2 ± 2.6 ^1^	11.8 ± 2.1 ^1^	
	CHO	11.2 ± 2.6	11.5 ± 2.6	12.0 ± 2.6	
RS at 4 mmol·L^−1^ [km·h^−1^]	PER	12.3 ± 2.1	13.5 ± 2.0	13.7 ± 1.7	0.094 (0.169)
	LCHF	12.1 ± 2.1	13.2 ± 2.2	13.9 ± 1.6	
	CHO	13.6 ± 2.2	14.1 ± 2.1	14.3 ± 2.1	

Bold numbers represent significant p-values. MFO, maximum fat oxidation; Fat max, intensity at MFO; TEE, total energy expenditure; RS, running speed. ^1,2,3^ significant difference between the timepoints during the intervention: ^1^compared to T-0, ^2^compared to T-1, ^3^compared to T-2. ^a,b,c^ significant difference between groups during the intervention: ^a^compared to PER, ^b^compared to LCHF, ^c^compared to CHO.

#### 3.6.1 Total fat oxidation

Total fat oxidation increased significantly from T-0 to T-1 in both the PER and the LCHF group (*p* = 0.007, *p* < 0.001, respectively). In the LCHF group no further changes were observed from T-1 to T-2 (*p* > 0.05), whereas fat oxidation decreased again in the PER group during this period (*p* = 0.018). At T-1, total fat oxidation was significantly higher in PER and LCHF groups compared with the CHO group (*p* = 0.009, ηp^2^ = 0.358). At the end of the intervention (T-2), total fat oxidation was significantly higher in LCHF group compared with both the PER and CHO groups (*p* = 0.004, ηp^2^ = 0.404). No significant changes over time were observed in the CHO group.

#### 3.6.2 Maximal fat oxidation (MFO)

A similar pattern was observed for the MFO. In the PER group, MFO increased significantly from T-0 to T-1 (*p* = 0.007) and decreased again from T-1 to T-2 (*p* = 0.006). In the LCHF group, MFO increased significantly from T-0 to T-1 (*p* < 0.001) and remained unchanged until T-2 (*p* > 0.05). At T-1, MFO was significantly higher in PER and LCHF groups compared to CHO group (*p* < 0.001, ηp^2^ = 0.564). At T-2, MFO was significantly higher in the LCHF group compared with PER or CHO groups (*p* < 0.001, ηp2 = 0.562). No significant changes in MFO were observed in the CHO group.

#### 3.6.3 Total carbohydrate oxidation, fat max and total energy expenditure

Total carbohydrate oxidation (*p* > 0.05, ηp^2^ = 0.115), and fat max (i.e., the intensity at which MFO occurs) did not show significant group x time interactions or main effects (*p* > 0.05). Total energy expenditure (TEE) increased significantly from T-0 to T-2 in the LCHF group (*p* = 0.023), with no significant differences between groups.

#### 3.6.4 Substrate oxidation at lactate threshold (LT)

CHO and fat oxidation at LT, expressed as percentage of the TEE, showed significant time x group interaction effects (*p* < 0.001, ηp^2^ = 0.441, respectively, Figure 4). In the PER group, CHO oxidation decreased and fat oxidation increased from T-0 to T-1 (*p* = 0.010, respectively), followed by a reversal of this pattern from T-1 to T-2, with CHO oxidation increasing and fat oxidation decreased (*p* = 0.013, respectively). In the LCHF group, CHO oxidation decreased and fat oxidation increased from T-0 to T-1 (*p* = 0.003), with no further changes observed until T-2. No significant changes were observed in the CHO group.

At T-1, CHO oxidation at LT was significantly different between all groups (*p* < 0.001, ηp^2^ = 0.629), with the highest CHO oxidation observed in the in CHO group, followed by the PER and LCHF groups. Conversely, fat oxidation at LT was the highest in LCHF group, followed by the PER and CHO groups (*p* < 0.001, ηp^2^ = 0.629). At the end of the intervention, CHO oxidation at LT was significantly lower and fat oxidation significantly higher in the LCHF group compared with both the PER and CHO groups (*p* < 0.001, ηp^2^ = 0.672, respectively).

#### 3.6.5 Lactate-related parameters

For total lactate concentration, running speed at LT, D_max mod_ and 4 mmol·L^−1^ no significant time x group interaction effects were observed (Table 4). However, all parameter showed significant main effects of time (*p* < 0.05). Running speed at 2 mmol·L^−1^ increased significantly from T-0 to T-1 and T-2 in both the PER and LCHF groups (*p* < 0.05).

## 4 Discussion

This study examined the effects of a periodized carbohydrate (CHO) intake strategy aligned with training phases on physiological responses during a graded exercise test, and body composition in recreationally active males over a period of 8 weeks. While no significant time x group interactions were observed for performance related outcomes, the intervention elicited distinct changes in substrate utilization and body composition between dietary groups. These findings suggest that nutritional periodization primarily influenced metabolic adaptations rather than performance outcomes assessed under laboratory conditions.

During the initial 4 weeks, characterized by basic endurance training combined with low CHO availability in the PER and LCHF groups, both groups demonstrated increases in total fat oxidation and fat oxidation at the lactate threshold. These results align with previous research indicating that reduced CHO availability enhances reliance on fat metabolism, likely mediated by increased free fatty acid availability and associated enzymatic adaptations (39–42). In contrast, no significant changes in substrate metabolism were observed in the CHO group, which maintained a high CHO intake throughout the study.

Conversely, in the latter half of the study, where the training intervention shifted toward more intense training sessions and CHO intake was reintroduced in the PER group, CHO oxidation at LT and total CHO oxidation returned to baseline levels - although total CHO oxidation barely missed statistical significance (*p* = 0.063) - accompanied by a normalization of fat oxidation. This finding suggests a restoration of metabolic flexibility, defined as the capacity to efficiently switch between substrates depending on availability and demand (43). The persistence of reduced CHO oxidation at the lactate threshold in the LCHF group supports previous evidence that prolonged CHO restriction may impair carbohydrate utilization through reduced glycogenolysis and pyruvate dehydrogenase activity (44–46). Notably, these results indicate that metabolic adaptations induced by a LCHF diet may require several weeks of CHO repletion to fully reverse, consistent with recent findings by Burke et al. (47).

Despite these metabolic alterations, no significant differences were observed between groups for V̇O₂peak, peak running speed, or time to exhaustion. *Post hoc* power analyses were conducted to aid interpretation of these non-significant findings and revealed limited statistical power for detecting group x time interaction effects for peak running speed (0.59) and time to exhaustion (0.75), whereas power was adequate for relative V̇O₂peak (0.89). Therefore, the absence of significant interaction effects for some performance outcomes - particularly PRS and TTE - should be interpreted with caution, as limited power may have contributed to type II error. Nevertheless, the lack of change in V̇O₂peak across groups is consistent with previous studies in recreationally trained individuals, where short- to medium-term dietary interventions without substantial differences in training load rarely elicit changes in maximal oxygen uptake (20). The absence of changes in both relative and absolute V̇O₂peak suggests that the observed reduction in body mass did not confound the interpretation of maximal oxygen uptake.

With respect to lactate-derived outcomes, no group differences were observed for lactate thresholds. However, running speed at a fixed lactate concentration of 2 mmol·L^−1^ increased in the PER and LCHF groups during the first 4 weeks and remained elevated thereafter. As running speed at low lactate concentrations is considered indicative of aerobic capacity (48), these results suggest that endurance training adaptations were preserved irrespective of subsequent dietary changes (i.e., restored CHO intake). However, it should be acknowledged that the observed increase in running speed at a fixed blood lactate concentration of 2 mmol·L^−1^ in the PER and LCHF groups may not solely reflect improvements in aerobic capacity. Reduced carbohydrate availability has been shown to attenuate glycolytic flux and lactate production, as well as to influence lactate efflux and buffering capacity. Consequently, lower blood lactate concentrations at a given running speed may result in a rightward shift of the lactate-speed curve, leading to higher speeds at fixed lactate thresholds independent of true aerobic adaptations. Notably, this altered lactate response appeared to persist even after carbohydrate reintroduction in the PER group, suggesting that dietary effects on lactate kinetics may extend beyond the immediate phase of carbohydrate restriction. Similar observations have been reported in previous studies investigating low-carbohydrate or ketogenic dietary conditions (47, 49).

Interestingly, running economy at LT improved only in the PER group following the reintroduction of a CHO-rich diet. This observation may reflect improved efficiency of CHO oxidation during higher-intensity exercise, as CHO provides a greater ATP yield per unit of oxygen compared to fat (50). Several studies have reported impaired exercise economy following LCHF diets, likely due to increased oxygen cost associated with predominant fat oxidation (51, 52). Thus, the present findings suggest that while LCHF nutrition enhances fat oxidation, reintroduction of CHO may be necessary to optimize exercise efficiency during intensified training phases. It should be acknowledged that running economy was assessed at the individual lactate threshold speed at each time point rather than at a fixed absolute running speed. To address this concern, running economy was additionally analyzed at a fixed running speed of 7.5 km·h^−1^ across all time points. Although the group x time interaction narrowly missed statistical significance, descriptive trends indicated a reduction in oxygen cost over time in the PER group, whereas no consistent changes were evident in the LCHF or CHO groups. This suggests that improvements in running economy in the PER group were not solely attributable to increased lactate threshold velocity, but may also reflect genuine adaptations in exercise efficiency. Nevertheless, given the well-established increase in oxygen cost associated with elevated fat oxidation, particularly under low-carbohydrate conditions, and previous reports of impaired running economy following LCHF diets, the present findings should be interpreted cautiously. Future studies should prioritize fixed-speed assessments of running economy to more clearly disentangle metabolic adaptations from changes in exercise intensity.

Regarding the changes in body composition, favorable changes were observed during the initial 4 weeks in the LCHF and PER group. Body weight and fat mass were further reduced in the latter 4 weeks in the LCHF group, whereas no additional significant change was observed in the PER group. In addition to its effects on substrate metabolism, a LCHF diet is frequently recommended for athletes due to its rapid weight reduction, which may be particularly beneficial in sports such as weightlifting or combat sports (53, 54). These effects may partly be explained by glycogen depletion (53) and associated water loss (55), as well as reduced energy intake and appetite commonly reported during LCHF diets (13, 56). Importantly, reintroduction of CHO in the PER group did not result in significant weight regain, suggesting that short-term LCHF phases do not necessarily induce a rebound effect when CHO intake is restored. In addition, the consistently low reported energy intake may have contributed to the observed changes in body composition independently of macronutrient distribution, and should therefore be considered when interpreting these outcomes.

The nutritional intervention was designed on a free-living, ad-libitum diet to ensure the comparability of the athlete’s real-life conditions. However, a key limitation of the present study is the assessment of dietary intake via self-reported food logs. Although dietary intake was monitored through regular screenshot submissions and post-study data exports from the FDDB application to enhance completeness and plausibility, objective verification of actual food intake was not possible. Consistent with previous research (57), under-reporting - particularly of total energy intake - was evident, as reflected by the unusually low reported energy intake across groups. This systematic bias may have affected estimates of energy availability and, consequently, influenced changes in body composition. Therefore, dietary intake data and related outcome measures should be interpreted with caution. Future studies should consider incorporating more objective dietary assessment methods, such as doubly labeled water or controlled feeding protocols, to improve data validity. Furthermore, with less than 50% of total energy intake from CHO, at any time point not all of the recreational active subjects were able to meet the recommended amount of 45–65% for the general population (58). On average, the subject ingested 2.8 ± 4.7 g·kg BW^−1^ prior to the study, 2.7 ± 6.7 g·kg BW^−1^ in the PER, and 3.6 ± 7.9 g·kg BW^−1^ in the CHO group – being at the lower end of the recommendations for recreational active athletes (59–61). Notably, after transitioning from a LCHF diet, it seemed even more challenging to achieve the recommended intake of CHO. However, it should be noted that the CHO intake below the recommendations was not only observed in the present sample, but also in other athlete cohorts (62–64).

Finally, the present study was conducted in recreationally active males and assessed physiological responses during a graded exercise test rather than discipline-specific performance outcomes such as time trials. While time to exhaustion provides insight into endurance capacity, it does not directly translate to real-world or competitive performance. Consequently, the findings should be interpreted within the context of laboratory-based physiological outcomes. Although running economy was primarily assessed at the velocity corresponding to the individual lactate threshold, this approach may be influenced by changes in threshold speed over time. While an additional fixed-speed analysis was included to address this concern, results should still be interpreted with caution when comparing running economy across time points. The study’s strengths include its rigorous research methods, the homogeneity of the athletes, and the measurement of outcomes following two distinct training periods. The study’s methodology and results offer novel insights into the field of sports nutrition, particularly the complementary nutritional and training interventions over two mesocycles in endurance athletes, which provide new insights into the effects of nutritional periodization on substrate utilization and body composition.

## 5 Conclusion

In conclusion, the present study demonstrates that periodized carbohydrate intake aligned with training phases influences substrate utilization, running economy, and body composition in recreationally active males, without inducing clear performance enhancements in a graded exercise test. Low-carbohydrate availability during base training increased fat oxidation and reduced carbohydrate utilization, whereas reintroduction of carbohydrates restored metabolic flexibility and improved running economy in the PER group. No additional metabolic benefits were observed with prolonged low-carbohydrate intake beyond 4 weeks. Importantly, the observed changes in running economy should not be interpreted as direct improvements in performance.

Although peak running speed and time to exhaustion improved over time, these changes were attributable to training adaptations rather than dietary group differences. Due to limited statistical power for some interaction effects and the absence of performance-based assessments such as time trials, no conclusions can be drawn regarding real-world or competitive performance. Overall, the findings highlight that nutritional periodization primarily affects metabolic and body composition variables rather than performance indicators and should therefore be interpreted cautiously. Further research using adequately powered designs and performance-specific outcomes is required to clarify the long-term implications of nutritional periodization for athletic performance.

## Data Availability

The raw data supporting the conclusions of this article will be made available by the authors, without undue reservation.

## Footnotes

1
https://doi.org/10.17605/OSF.IO/VM7KB
