# Supplementary material for: Periodized carbohydrate intake influences metabolic flexibility and indices of running economy during endurance training in recreationally active males
Source: Front Nutr. 2026 Jan 15;12:1750042. doi: 10.3389/fnut.2025.1750042 (PMC12851979; doi:10.3389/fnut.2025.1750042)
Supplement: Supplementary file 1 [file Data_Sheet_1.pdf]

NUTRITIONAL GUIDELINES

	**LCHF** or **PER** (week 1-4)	**CHO** or **PER** (week 5-8)
**General information**<br>**Carbohydrates**	≤ 50 g per day	50-55%
**Fat (65% plant-based and unprocessed fats such as oil, fish, nuts and seeds)**	≥ 65%	25%
**Proteins**	15 – 20%	15 – 20 %
**Meat, fish and eggs**	No restrictions except for breaded products<br>*Examples: steak, salmon, chicken breast, eggs, cold cuts*	No restrictions, but in small quantities and preferably low-fat varieties<br>*Examples: steak, salmon, chicken breast, eggs, cold cuts*
**Vegetables**	Low-starch vegetables such as cabbage, cucumbers, lettuce, tomatoes and peppers<br>Starchy vegetables such as potatoes or sweet potatoes should be avoided	No restrictions
**Fruits**	Maximum daily amount of 200g<br>Low-sugar fruits such as berries, papayas and watermelon are permitted	No restrictions
**Dairy**	No fresh dairy products (milk, some low-fat yoghurts and fruit yoghurts) due to their high carbohydrate content<br>Full-fat cheese, whole milk yoghurt and low-carbohydrate cream cheese are permitted	No restrictions, but low-fat varieties should be preferred<br>Mature cheese with a high fat content should only be consumed occasionally
**Cereal products**	Not permitted due to their high carbohydrate content<br>Low-carbohydrate breads made from nuts and coconut or almond flour can replace conventional bread	White flour products and wholemeal products<br>Examples: white bread, toast, white rice, millet, couscous, bulgur, pasta and potatoes
**Nuts and seeds**	No restrictions except for chestnuts and cashews	No restrictions, but in small quantities (fat source)
**Beverages**	Coffee, unsweetened teas and water<br>No alcoholic beverages, soft drinks and juices	No restrictions on non-alcoholic beverages<br>No alcoholic beverages
**Sweeteners, sweets and snacks**	Nuts, berries and dark chocolate (≥ 70% cocoa), cinnamon,sugar substitutes such as stevia or xylitol are permitted	Low-fat snacks such as gummy bears, sorbets, rice cakes, low-fat crisps and sports bars, sugar
