# Supplementary material for: Periodized carbohydrate intake influences metabolic flexibility and indices of running economy during endurance training in recreationally active males
Source: Front Nutr. 2026 Jan 15;12:1750042. doi: 10.3389/fnut.2025.1750042 (PMC12851979; doi:10.3389/fnut.2025.1750042)
Supplement: Supplementary file 2 [file Data_Sheet_2.pdf]

Supplementary Material

Training plan

Training intensity zones were defined individually for each participant based on the results of the graded exercise test. The regeneration zone (REG) represented very low-intensity exercise below the first lactate threshold. GA1 corresponded to exercise intensity around the first lactate threshold (LT1). GA1–2 described the intensity range between the first and second lactate thresholds. GA2 represented intensities around the second lactate threshold, whereas WSA (Wettkampfspezifischer Ausdauerbereich) reflected maximal or near-maximal intensity exercise. To ensure realistic and individualized training prescription, heart rate zones were adjusted for each participant, with an approximate range of 10–12 heart beats per minute per zone.

**training week 1**		
**Training**	**description**	**duration (min)**
Base endruance run	GA1	40
Intervals	10:00 Warm-up<br>5x (0:45 GA2 + 1:15 rest)<br>10:00 Cool down	30
Base endruance run	REG	30
Intervals	5:00 Warm-up<br>25:00 Fartlek in GA1-2	30
Long Run	GA1 - GA1-2	60
**training week 2**		
**Training**	**description**	**duration (min)**
Base endruance run	GA1	45
Intervals	10:00 Warm-up<br>3x (1:00 WSA + 1:00 rest)<br>3x (2:00 GA2 + 1:00 rest)<br>10:00 Cool down	35
Base endruance run	REG	35
Intervals	5:00 Warm-up<br>35:00 Fartlek in GA1-2	40
Long Run	GA1 - GA1-2	80
**training week 3**		
**Training**	**description**	**duration (min)**
Base endruance run	GA1	50
Intervals	10:00 Warm-up<br>1x (1:30 GA2 + 1:00 rest)<br>3x (0:30 WSA + 1:00 rest)<br>1x (1:30 GA2 + 1:00 rest)<br>3x (0:30 WSA + 1:00 rest)<br>10:00 Cool down	35

Base endruance run	REG	40
Intervals	5:00 Warm-up<br>20:00 abwechselnd 1:00 GA1-2 + 1:00 REG	25
Long Run	GA1 - GA1-2	100
**training week 4**		
**Training**	**description**	**duration (min)**
Base endruance run	GA1	30
Intervals	10:00 Warm-up<br>10x (0:30 WSA + 1:00 rest)<br>10:00 Cool down	35
Base endruance run	REG	30
GXT		
**training week 5**		
**Training**	**description**	**duration (min)**
Base endruance run	GA1	45
Intervals	10:00 Warm-up<br>10x (02:00 GA2 + 1:00 rest)<br>10:00 Cool down	50
Base endruance run	REG	35
Intervals	10:00 Warm-up<br>5:00 GA2 + 2:00 rest<br>10:00 GA1-2 + 3:00 rest<br>15:00 GA1<br>5:00 Cool down	50
Long Run	GA1 - GA1-2	100
**training week 6**		
**Training**	**description**	**duration (min)**
Base endruance run	GA1	50
Intervals	10:00 Warm-up<br>3x (1x (3:00 GA2 + 2:00 rest) + 4x (0:30 WSA + 1:00 rest))<br>10:00 Cool down	55
Base endruance run	REG	40
Intervals	10:00 Warm-up<br>30:00 GA1-2<br>5:00 Cool down	45
Long Run	GA1 - GA1-2	110
**training week 7**		
**Training**	**description**	**duration (min)**
Base endruance run	GA1	50

Intervals	10:00 Warm-up<br>3x (5:00 GA1-2 + 2:00 rest)<br>5:00 Cool down	36
Base endruance run	REG	40
Intervals	5:00 Warm-up<br>25:00 Fartlek mit 2:00 GA2 + 1:00 REG	30
Long Run	GA1 - GA1-2	75
**training week 8**		
**Training**	**description**	**duration (min)**
Base endruance run	GA1	30
Intervals	10:00 Warm-up<br>2x (1:30 GA2 + 1:30 rest + 1:30 GA1-2 + 1:30 rest + 1:30 WSA + 1:30 rest)<br>5:00 Cool down	35
Base endruance run	REG	30
GXT		
